# Effect of anticoagulants on fibrin clot structure: A comparison between vitamin K antagonists and factor Xa inhibitors

**DOI:** 10.1002/rth2.12443

**Published:** 2020-10-25

**Authors:** Julia S. Gauer, Nicoletta Riva, Eden M. Page, Helen Philippou, Michael Makris, Alex Gatt, Robert A. S. Ariëns

**Affiliations:** ^1^ Discovery and Translational Science Department Institute of Cardiovascular and Metabolic Medicine University of Leeds Leeds UK; ^2^ Department of Pathology Faculty of Medicine & Surgery University of Malta Msida Malta; ^3^ Sheffield Haemophilia and Thrombosis Centre University of Sheffield Sheffield UK

**Keywords:** anticoagulants, factor Xa inhibitors, fibrin clot, LMWH, warfarin

## Abstract

**Background:**

Abnormal clot structure has been identified in patients with thrombotic disorders. Anticoagulant therapy offers clear benefits for thrombosis prevention and treatment by reducing blood clot formation and size; nevertheless, there are limited data on the effects of different anticoagulants, where clotting is initiated with different triggers, on clot structure.

**Objectives:**

Our aim was to investigate the effects of vitamin K antagonists and factor Xa inhibitors on clot structure.

**Methods:**

Clots from pooled plasma spiked with rivaroxaban, apixaban, or enoxaparin, as well as plasma from patients on warfarin, were compared to plasma without anticoagulation. The kinetic profile of polymerizing clots was obtained by turbidity, fiber density was determined by confocal microscopy, clot pore size was investigated by permeation, and fiber size was analyzed using scanning electron microscopy. Clotting agonist was either tissue factor or thrombin.

**Results:**

Following clotting with tissue factor, all anticoagulated clots had a significantly increased lag time, with the exception of enoxaparin. Rivaroxaban additionally led to significantly less dense and more permeable clots, with thicker fibers. In contrast, turbidity analysis following initiation with thrombin showed few effects of anticoagulation, with only enoxaparin leading to a prolonged lag time. Enoxaparin clots made with thrombin were less dense and more permeable.

**Conclusion:**

Our results show that anticoagulants modulate clot structure particularly when induced by tissue factor, most likely due to reduction of thrombin generation. We propose that the effects of different anticoagulants could be assessed with a global clot structure measurement such as permeation or turbidity, providing information on clot phenotype.

**Essentials**

- Anticoagulant therapies are associated with different bleeding risks.
- There are limited data on the effects of anticoagulants on fibrin clot structure.
- Initiation with tissue factor but not thrombin reveals effects of direct oral anticoagulants on clot structure.
- Clot structure could be used as a global test for anticoagulant activity.

## 1 INTRODUCTION

Thrombosis is a major contributor to global disease burden, being a serious and lethal component of cardiovascular diseases such as ischemic heart disease, stroke, and venous thromboembolism.^1^, ^2^ Thromboembolic conditions accounted for one in four deaths worldwide in 2010 and remain the leading cause of death around the world.^3^, ^4^, ^5^, ^6^ Anticoagulants decrease blood clot formation by targeting and modulating the coagulation pathway and are, therefore, commonly administered to prevent and treat thromboembolic disorders.^7^ However, all anticoagulant drugs to date are associated with a certain bleeding risk that needs to be considered in the decision of anticoagulation administration.^8^, ^9^

Conventionally, individuals with thrombosis received a combination of low‐molecular‐weight heparins (LMWHs), followed by vitamin K antagonists (VKAs), particularly warfarin. Direct oral anticoagulants (DOACs), which directly inhibit thrombin or activated factor X (FXa), have more recently been developed. Unlike warfarin, DOACs can be administered at fixed doses, thus reducing the number of hospital visits for drug monitoring.^10^, ^11^

Previous studies have shown that fibrin clot structure is abnormal in individuals with thrombotic conditions, and individuals with with postthrombotic syndrome and recurrent venous thromboembolism (VTE) patients have denser clots with thinner fibers that are resistant to fibrinolysis.^12^ In addition, clots from individuals with deep vein thrombosis were also shown to be thinner, less porous, and more resistant to lysis.^13^ These studies indicate that abnormal fibrin clot structure is associated with patients with thrombotic disorders. Anticoagulants, including warfarin and DOACs, have been shown to alter fibrin clot structure by increasing clot porosity and altering clot density.^11^, ^14^, ^15^ Direct thrombin inhibitors have also been shown to delay thrombin generation and increase clot permeability.^16^, ^17^ In addition, anticoagulants have been shown to delay clotting lag time and increase the efficiency of clot lysis.^11^, ^18^, ^19^, ^20^, ^21^

The effects of anticoagulants on clot structure have been previously investigated using thrombin^12^, ^13^, ^15^, ^22^ or tissue factor (TF),^11^, ^14^, ^16^, ^17^, ^19^, ^20^, ^21^, ^23^, ^24^ as well as TF‐bearing cells^25^, ^26^ as clotting agonists. In addition, thrombus growth and fibrin distribution patterns, related to clot stability, have also been studied with TF‐bearing collagen surfaces.^27^, ^28^ TF has been reported to better reflect effects of VKAs on clot density and permeability.^29^ In this study, we compared clot structure in plasma from individuals on warfarin, pooled plasma (PP) spiked with the direct FXa inhibitors rivaroxaban and apixaban, and PP spiked with the indirect FXa inhibitor enoxaparin, to PP control using a range of sensitive methods that analyze clot density, porosity, and fiber thickness. We thus used a comprehensive set of clot structure analysis methods to directly compare the effects of VKAs and indirect/direct FXa inhibitors on fibrin clot structure when clotting was triggered with either thrombin or TF.

## 2 MATERIALS AND METHODS

### 2.1 Materials

Alexa Fluor 594–fibrinogen (Thermo Fisher Scientific, Altrincham, UK) was reconstituted in double‐distilled water (ddH_2_O) to 2.5 mg/mL. Human thrombin (Merck Diagnostics, Darmstadt, Germany) was reconstituted to 250 U/mL in ddH_2_O and stored at –80°C. Dilutions were performed in 0.05M Tris‐Base, 0.1 M NaCl, pH 7.4 (TBS). PPP‐Reagent (Stago, Parsippany, NJ, USA) was reconstituted in 1 mL of ddH_2_O to 30 pM of TF and 24 µM phospholipids. All other chemicals were obtained from Sigma‐Aldrich (St. Louis, MO, USA) unless stated otherwise.

Apixaban and rivaroxaban powder (MedChemExpress, Princeton, NJ, USA) were initially reconstituted in dimethyl sulfoxide and subsequently diluted 1:9 in deionized water. They were spiked in normal‐pooled plasma (NPP) at concentrations of apixaban 128 ng/mL and rivaroxaban 174 ng/mL both at therapeutic levels. Enoxaparin sodium (Clexane) was spiked in NPP at concentration 0.35 U/mL, that is, equivalent to a prophylactic dose.

### 2.2 Blood collection

A pool of plasma from 355 outpatient clinic (preoperative assessment) patients with normal international normalized ratio (INR)/activated partial thromboplastin time was obtained from anonymized normal citrated samples (Vacuette, Greiner Bio‐One, Monroe, NC USA) and analyzed in the Coagulation Laboratory at Mater Dei Hospital (Msida, Malta). As our pooled plasma was obtained from individuals undergoing a wide range of surgical procedures, it includes a mixture of individuals with and without underlying health conditions. All samples underwent double centrifugation (10 minutes at 2500 *g* twice, using the Eppendorf Centrifuge 5810, Eppendorf AG, Germany) to obtain platelet‐poor plasma (PPP), and they were frozen at −80°C. They were subsequently spiked with apixaban, rivaroxaban, and enoxaparin at the desired concentrations. Warfarinized PPP was collected from anonymized outpatients’ samples receiving warfarin therapy. There were two pools of warfarinized plasma, that is, INR 2.2 (samples of patients with INR 2‐3) and INR 4.1 (samples from patients with INR 4‐6) to represent therapeutic and supratherapeutic INR, respectively. They were prepared following the same protocol of the plasma spiked with anticoagulants and underwent an identical number of freeze‐thaw cycles. Frozen anticoagulated samples were shipped to Leeds and Sheffield on dry ice. Pooled plasma (PP) with a measured fibrinogen concentration of 4.64 mg/mL was used as reference. PP from individuals on warfarin INR 2.2 (3.80 mg/mL fibrinogen) and plasma from individuals on warfarin INR 4.1 (4.31 mg/mL fibrinogen) were chosen, as their fibrinogen levels were most similar to PP.

The prothrombin time/INR assay, fibrinogen concentrations, and the anti‐Xa assay for enoxaparin were performed using an ACL TOP 500 analyzer (Instrumentation Laboratory, Bedford, MA, USA) and the following reagents: HemosIL RecombiPlasTin 2G, HemosIL QFA Thrombin (Bovine), and HemosIL Liquid Anti‐Xa kit (Instrumentation Laboratory). The anti‐Xa assays for apixaban and rivaroxaban were performed in the Coagulation Laboratory at the Royal Hallamshire Hospital, using the analyser Sysmex CS‐5100 (Siemens Healthcare Diagnostics Products GmbH, Marburg, Germany) and the Biophen DiXal kit (Hyphen BioMed, Neuville‐sur‐Oise, France) with specific calibrators.

Finally, normal pooled platelet‐poor plasma (NPP) used in some of the supplementary experiments was obtained from 30 healthy individuals at the Leeds Institute of Cardiovascular and Metabolic Medicine, with a fibrinogen concentration of 3.4 mg/mL.

### 2.3 Thromboelastography

Thromboelastography (TEG) was performed at Mater Dei Hospital (Msida, Malta) on citrated PPP, using a TEG5000 (Thromboelastograph Hemostasis Analyser, Haemoscope, Haemonetics Corporation, Braintree, MA, USA) with dedicated software (TEG Analytical Software version 4.2.3, Haemoscope, Haemonetics Corporation).

The native TEG used citrated PPP 330 µl and CaCl_2_ 0.2M 30 µl (without the addition of a clotting trigger such as thrombin or TF) and was performed in duplicate, using the two channels provided. The results were expressed as mean ± SD.

### 2.4 Turbidity analysis of polymerizing fibrin clots

The kinetic profile of polymerizing fibrin clots was investigated by turbidity, using a standardized protocol as previously described.^30^ In brief, plasma was diluted to a final ratio of 1 in 6 in TBS and loaded onto 384‐well plates (Thermo Fisher Scientific, Altrincham, UK) in triplicates. An activation mixture of 5 mM CaCl_2_ (final concentration) and either tissue factor (final concentration, 1 pM) or thrombin (final concentration, 0.1 U/mL) was added to initiate clotting. Thrombin concentration was chosen following optimization for this and other methods described. TF concentration used in all methods was chosen to reflect the low TF concentration used in the calibrated automated thrombogram thrombin generation assay. Changes in absorbance (or optical density) were measured at 340 nm every 17 seconds for 1 hour at 37°C using a Powerwave microtiter‐plate reader (Bio‐Tek, Swindon, UK). The parameters analyzed from the turbidity profile were lag time to initiation of polymerization, maximum absorbance (an indicator of fiber diameter) and average rate of clotting (based on the rate between 25% and 75% clotting). Turbidity experiments were performed in duplicate or triplicate, each with three technical replicates.

### 2.5 Laser scanning confocal microscopy

Plasma was diluted to a final ratio of 1 in 6 with TBS and spiked with Alexa Fluor 594 labeled fibrinogen at 50 µg/mL final concentration. Clotting was triggered by the addition of 5 mM CaCl_2_ (final concentration) and either TF (final concentration, 1 pM) or thrombin (final concentration, 0.1 U/mL). Once clotting was initiated, the mixture was immediately transferred to the channel of an uncoated µ‐Slide VI 0.4 mm (Ibidi GmbH, Gräfelfing, Germany), and placed in a dark humidity chamber for 2 hours at room temperature to allow clots to form. Images were obtained using a Zeiss LSM880 inverted microscope with a 40× oil immersion lens. Optical z‐stacks (43 × 0.7 µm) were combined and flattened to show maximum intensity. Fiber density was determined by averaging the total number of fibers crossing an arbitrary straight line of fixed length (200 µm). Image processing and fiber counting were executed on ImageJ software. Fibrin clots were prepared in triplicate, and three density measurements were obtained per clot.

### 2.6 Clot permeation

The clot permeability assay was adapted from a previously described method.^31^ Plasma samples were diluted to a final ratio of 1 in 8.8 with TBS. Clotting was initiated by the addition of an activation mixture containing 10 mM CaCl_2_ (final concentration) and either TF (final concentration 1 pM) or thrombin (final concentration, 0.5 U/mL). Immediately after initiation of clotting, the mixture was transferred to the channel of an Ibidi uncoated µ‐Slide VI 0.4 mm, then placed in a humidity chamber for 1 hour at room temperature to allow clots to form. A plastic syringe was connected to the well corresponding to the channel holding the clot, and a constant pressure was applied by filling the syringe with TBS to a set height (4 cm). The clots were washed in this manner for 30 minutes, and flow rates of buffer through the clots were then measured, by collecting and weighing the buffer flow‐through, every 10 minutes for 40 minutes. Volume (correlated to weight, assuming 1g = 1mL) of flow‐through over time was plotted and fitted by linear regression (R^2^ ≥ 0.99). The permeation coefficient (K_s_, Darcy constant) was calculated as previously described.^32^ The slightly higher plasma dilution factor, thrombin, and calcium concentrations were required to obtain a measurable flow rate in the permeation assay. Permeation experiments were performed in triplicate or quadruplicate.

### 2.7 Scanning electron microscopy

Clots for scanning electron microscopy (SEM) were prepared and imaged using a previously described method.^33^ In brief, plasma was diluted 1 in 6 with TBS, and clotting was triggered by the addition of an activation mixture consisting of 10 mM CaCl_2_ (final concentration) and either TF (final concentration, 1 pM) or thrombin (final concentration, 1 U/mL). Immediately following clotting initiation, the mixture was transferred to pierced Eppendorf lids and placed in a humidity chamber for 2 hours to allow for clot formation. Clots were washed 3 times for 40 minutes with saline solution and fixed in 2% glutaraldehyde overnight. Once fixed, clots were washed 3 times for 40 minutes with sodium cacodylate buffer (50 mM C_2_H_12_AsNaO_5_, pH 7.4) and dehydrated in increasing concentrations of acetone (30%‐100%). Clots underwent critical point drying with CO_2_ before being mounted onto stubs and sputter coated with 10 nm of iridium using a Cressington 208 HR (Cressington Scientific Instruments, Watford, UK). Each clot was formed in triplicate and imaged in three different areas at different magnifications, 5000‐40 000×) using a Hitachi SU8230 high‐performance cold field emission SEM (Chiyoda Corporation, Yokohoma, Japan). Fibrin fiber thickness was analyzed on images at 20 000× using ImageJ.

### 2.8 Statistical Analysis

Data are presented as mean of at least three replicates ± SE, unless otherwise stated. Differences between groups was determined by one‐way analysis of variance or Kruskal–Wallis test, following Shapiro–Wilk test for normality, followed by Tukey–Kramer post hoc test or Dunn–Bonferroni to determine significant differences to control group. Data analysis was performed using OriginPro 2017/2018. *P* values<.05 were considered to indicate statistical significance.

## 3 RESULTS

### 3.1 Thromboelastography

Clot formation in the absence of a clotting trigger was analysed by native TEG (Table 1). The average R time, reflective of the lag time before the start of clotting, was increased for PP spiked with the direct FXa inhibitor rivaroxaban (19.55 ± 0.07 minutes; *P* < .05) and enoxaparin (23.35 ± 0.35 minutes; *P* < .05), compared to control (12.97 ± 3.26 minutes). Clot formation time (K time) was also increased for PP spiked with direct FXa inhibitors (rivaroxaban, 7.50 ± 2.12 minutes; and apixaban, 6.00 ± 3.11 minutes; *P* < .05) and enoxaparin (11.40 ± 2.12 min; *P* < .05), compared to control (2.64 ± 0.55 minutes). The α‐angle corresponding to the tangent line angle from 2 mm to 20 mm amplitude was narrower for PP spiked with rivaroxaban (27.40 ± 7.92°; *P* < .05) and enoxaparin (17.40 ± 0.99°; *P* < .05), compared to control (52.2 ± 7.6°). We also observed a slight increase in maximum amplitude (MA), reflective of the ultimate clot strength, for both warfarinized plasma samples (INR, 2.2 37.80 ± 2.83 mm; INR, 4.1 38.05 ± 1.34 mm; *P* < .05) and a small reduction for PP spiked with rivaroxaban (28.40 ± 0.57 mm; *P* < .05) and enoxaparin (28.20 ± 0.99 mm; *P* < .05) relative to control (32.92 ± 1.86 mm).

**Table 1:** TEG results of analysis of recalcified plasma samples containing different anticoagulants, in the absence of clotting triggers

	R time (min)	K time (min)	α‐angle (deg)	MA (mm)
PP	12.97 ± 3.26	2.64 ± 0.55	52.47 ± 7.65	32.92 ± 1.86
Warfarinized PP INR 2.2	10.85 ± 1.34	2.80 ± 1.41	54.10 ± 15.84	37.80 ± 2.83*
Warfarinized PP INR 4.1	16.25 ± 6.01	3.75 ± 1.34	41.85 ± 10.82	38.05 ± 1.34*
PP + rivaroxaban (174 ng/mL)	19.55 ± 0.07*	7.50 ± 2.12*	27.40 ± 7.92*	28.40 ± 0.57*
PP + apixaban (128 ng/mL)	15.65 ± 4.45	6.00 ± 3.11*	36.95 ± 17.89	33.40 ± 6.65
PP + enoxaparin (0.35 U/mL)	23.35 ± 0.35*	11.40 ± 2.12*	17.40 ± 0.99*	28.20 ± 0.99*

Note

±SD.

Abbreviations: INR, international normalized ratio; MA, maximum amplitude; PP, pooled platelet‐poor plasma; TEG, thromboelastography.

* *P < *.05 (Mann‐Whitney nonparametric U test for the comparison with PP).

### 3.2 Clotting initiation with TF

**Figure 1:**
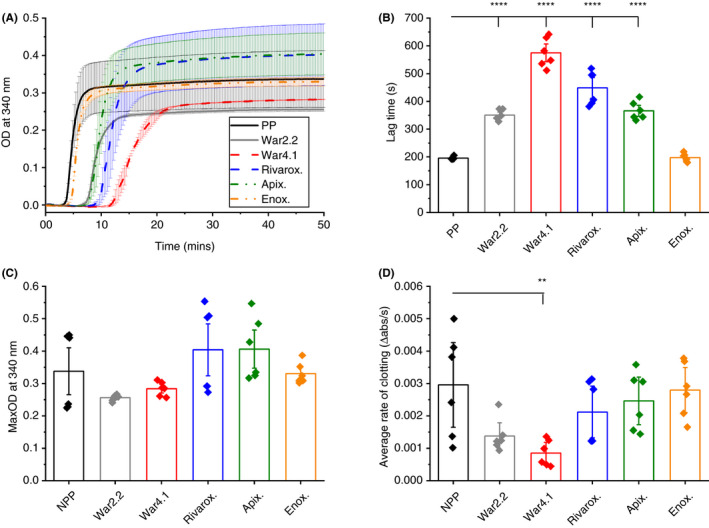
Turbidity analysis parameters of polymerizing clots with tissue factor. Clotting of diluted plasma samples was initiated with tissue factor and parameters of warfarinized plasma with international normalized ratio 2.2 (War2.2) and 4.1 (War4.1), and pooled plasma (PP) spiked with rivaroxaban (Rivarox.), apixaban (Apix.) or enoxaparin (Enox.) were compared against PP control. Turbidity curve of polymerizing clots; (A), error bars correspond to ± SE of two replicates, with three technical replicates. From turbidity curves clotting lag time (B), maximum absorbance (MaxOD) of fully polymerized clots (C), and average rate of clotting, defined by changes in absorbance over time (D), were determined. Data points correspond to individual technical replicates. Error bars correspond to ± SE. **P < .01, ****P < .0001. OD, optical density

Turbidity analysis of clots formed with TF showed clear differences in polymerization (Figure 1A). For instance, the lag time was significantly (*P* < .0001) longer for warfarinized plasma samples (2‐fold and 2.9‐fold for INR 2.2 and 4.1, respectively), and plasma spiked with rivaroxaban (2.3‐fold) and apixaban (1.8‐fold). The delay in clot formation for these samples suggests impaired protofibril formation and are in agreement with the longer K time obtained for FXa inhibitors in native TEG (Table 1). No change in lag time was observed for plasma spiked with enoxaparin (Figure 1B). Furthermore, there were no changes in maximum absorbance (MaxOD) for plasma samples containing anticoagulants compared to control (Figure 1C). Warfarinized plasma at INR 4.1 did, however, show a slower rate of clotting (3.5‐fold; *P* < .05) (Figure 1D). A slower average rate indicates that longer time was required for clots to achieve 75% clotting from 25%, where MaxOD equates to 100% clot formation. Time to 50% clotting and time to maximum rate of clotting (V_max_) follow the same pattern observed for lag time for these samples (Figure S1). A batch of (NPP from healthy individuals was spiked with higher concentrations of enoxaparin, equivalent to therapeutic dosage. Lag time for NPP + 0.6 U/mL enoxaparin was over 20 minutes, with neither NPP + 1 U/mL nor NPP + 2 U/mL clotting in 1 hour (Figure S2A).

### 3.3 Clotting initiation with thrombin

**Figure 2:**
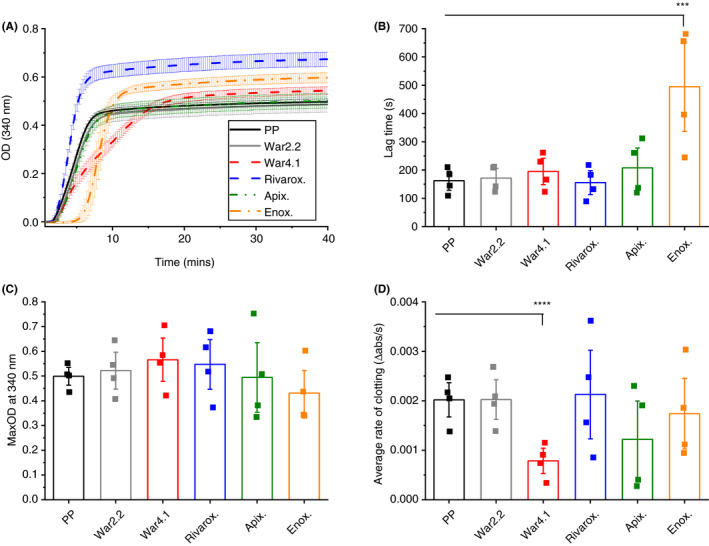
Turbidity analysis parameters of polymerizing clots with thrombin. Clotting of diluted plasma samples was initiated with thrombin and parameters of warfarinized plasma with international normalized ratio 2.2 (War2.2) and 4.1 (War4.1), and pooled plasma (PP) spiked with rivaroxaban (Rivarox.), apixaban (Apix.) or enoxaparin (Enox.) were compared against PP control. Turbidity curve of polymerizing clots; graph is a representation of one of four repeats (A), error bars correspond to ± SE of three technical replicates. From turbidity curves clotting lag time (B), maximum absorbance (MaxOD) of fully polymerized clots (C), and average rate of clotting, defined by changes in absorbance over time (D), were determined. Each data point represents the average of three technical replicates. Error bars correspond to ± SE. ***P < .001, ****P < .0001. OD, optical density

Turbidity analysis of polymerizing clots, when clotting was initiated with thrombin, showed relatively comparable curve patterns for plasma samples containing anticoagulants compared to control (Figure 2A). Interestingly, lag time was 3‐fold longer (*P* < .0001) for plasma samples spiked with enoxaparin compared to control (Figure 2B), suggesting impaired protofibril formation. Although LMWHs have reduced ability to inhibit thrombin due to their smaller size,^34^ the observed longer lag time for plasma containing enoxaparin after addition of thrombin clearly shows that this anticoagulant still has an effect on thrombin activity. This observation is also in agreement with the changes observed by native TEG for the enoxaparin clot (Table 1). Similarly to results discussed above where clots were formed with tissue factor, there were no changes in MaxOD for clots formed with thrombin (Figure 2C). Nevertheless, the average rate of clotting was 2.6‐fold (*P* < .0001) slower for warfarinized plasma at INR 4.1 (Figure 2D). Time to 50% clotting and V_max_ followed the same pattern observed for average rate of clotting and lag time for these samples (Figure S3). NPP spiked with 0.6‐2 U/mL enoxaparin produced no detectable clots (Figure S4).

### 3.4 Fiber density and permeability following clotting initiation with TF

**Figure 3:**
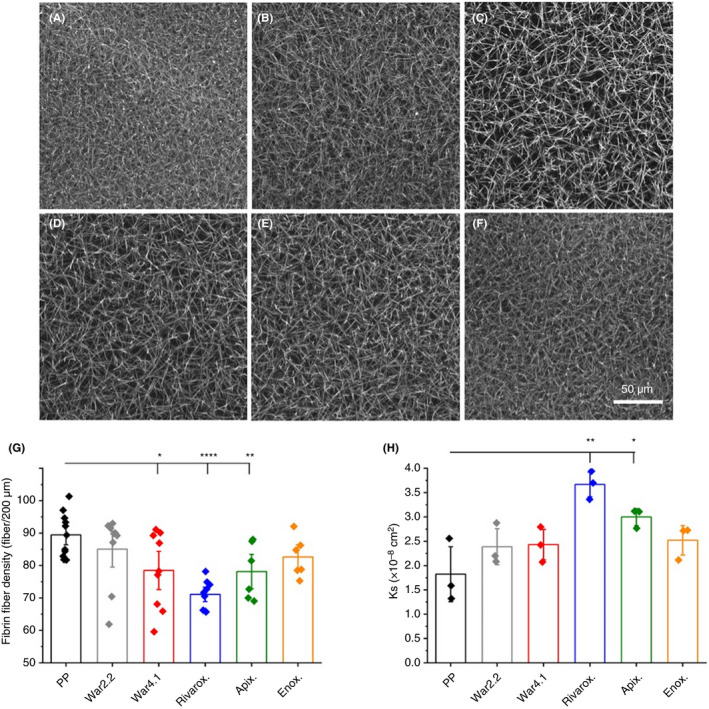
Fibrin fiber density and porosity of fully formed clots following clotting with tissue factor. Confocal microscopy images of pooled plasma (PP) (A), warfarin international normalized ratio (INR) 2.2 (B), warfarin INR 4.1 (C), PP + rivaroxaban (D), PP + apixaban (E), and PP + enoxaparin (F) were used to calculate fibrin fiber density (G). Images are a representation of one of three repeats, each imaged in three different areas of the clot. Each data point corresponds to individual measurements of three replicates. Scale bar represents 50 μm. Fibrin clot porosity (H) was determined by the permeation coefficient (K_s_), where higher K_s_ values indicate a more porous fibrin fiber network, and lower K_s_ values suggest a less porous fibrin fiber network. Each data point corresponds to one of three replicates. Error bars correspond to ± SE. *P < .05, **P < .01,****P < .0001

The structural architecture of fully formed clots, resulting from clotting initiation with tissue factor, was investigated using laser scanning confocal microscopy. There was a visual decrease in fiber density for warfarinized plasma at INR 4.1 and plasma containing rivaroxaban and apixaban (Figure 3C, D, and E, respectively) in comparison to control (Figure 3A). Moreover, average fiber number per 200 µm was decreased for these samples in relation to PP (89 ± 7), namely, warfarin INR 4.1 (78 ± 11, *P* < .05), rivaroxaban (71 ± 4, *P* < .0001), and apixaban (78 ± 7, *P* < .01) (Figure 3G). Correspondingly, the permeation coefficient (K_s_) was increased for clots of PP (1.8 ± 0.6 × 10^−8^ cm^2^) spiked with rivaroxaban (3.7 ± 0.3 × 10^−8^ cm^2^, *P* < .01) and apixaban (3.0 ± 0.2 × 10^‐8^ cm^2^, *P* < .05), indicating larger clot pore size for these samples (Figure 3H). Three‐by‐three tile scans show mostly homogeneity of the structure of clots (Figure S5). The decrease in fiber density and increase in porosity observed for rivaroxaban clots are in agreement with the decreased MA observed for rivaroxaban clots by native TEG (Table 1). Clots from plasma containing higher concentrations of enoxaparin, namely, 0.6 U/mL (7.3 ± 0.1 × 10^‐8^ cm^2^) and 1 U/mL (8.0 ± 0.2 × 10^−8^ cm^2^) also had increased (*P* < .05) pore size compared to control (3.7 ± 0.5 × 10^−8^ cm^2^) (Figure S2B). A higher K_s_ value was also observed for NPP containing 2 U/mL of enoxaparin (11.0 × 10^−8^ cm^2^; Figure S2B); however, only a single value was obtained as clots from further repeats did not withstand the constant flow. Confocal images of clots containing 0.6 U/mL enoxaparin confirmed a looser structure and decreased fiber number per 200 µm (36 ± 10) when compared to control (93 ± 2; *P* < .0001; Figure S2C), with higher concentrations of enoxaparin producing no visible clots.

### 3.5 Fiber density and permeability following clotting initiation with thrombin

**Figure 4:**
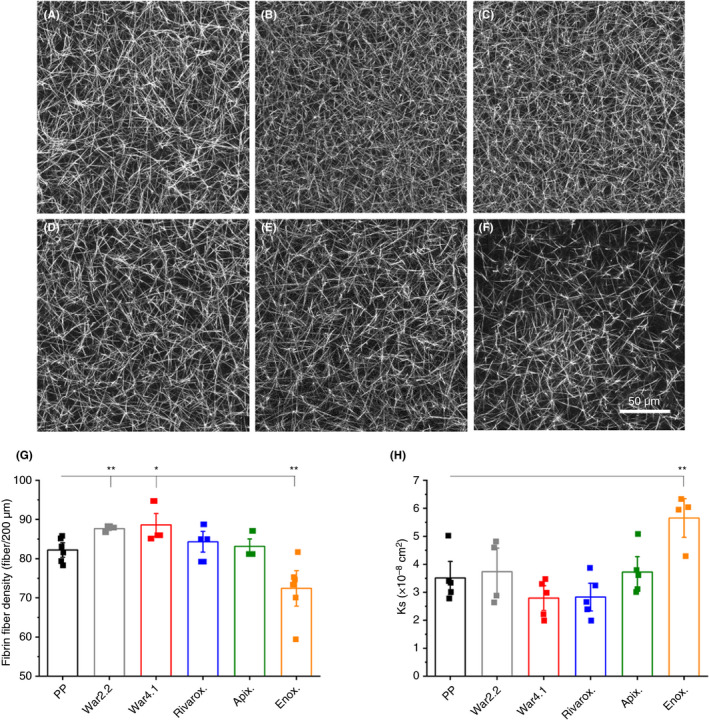
Fibrin fiber density and porosity of fully formed clots following clotting with thrombin. Confocal microscopy images of pooled plasma (PP) (A), warfarin international normalized ratio (INR) 2.2 (B), warfarin INR 4.1 (C), PP + rivaroxaban (D), PP + apixaban (E), and PP + enoxaparin (F) were used to calculate fibrin fiber density (G). Images are a representation of one of three repeats, each imaged in three different areas of the clot. Each data point corresponds to individual measurements of three replicates. Scale bar represents 50 μm. Fibrin clot porosity (H) was determined by the permeation coefficient (K_s_), where higher K_s_ values indicate a more porous fibrin fiber network, and lower K_s_ values suggest a less porous fibrin fiber network. Each data point corresponds to one of five replicates. Error bars correspond to ± SE. *P < .05, **P < .01

Fully formed clots produced with thrombin were also studied by laser scanning microscopy. Clots of warfarinized plasma (Figure 4B and C) were visually denser than control (Figure 4A). In addition, plasma spiked with enoxaparin formed visually less dense clots (Figure 4F). These visual observations correlated to the average fiber number in 200 µm, where warfarinized plasma samples, INR 2.2 (75 ± 2; *P* < .01) and INR 4.1 (77 ± 9; *P* < .05), had a higher average fiber count than PP (64 ± 6) and enoxaparin‐spiked plasma (45 ± 15; *P* < .01) (Figure 4G). Again, 3 × 3 tile scans showed homogeneity of all clots, excluding enoxaparin clots, which present a heterogeneous fibrin distribution (Figure S6). The fiber density follows the same trend as the MA results obtained by native TEG (Table 1). Moreover, clots of plasma containing enoxaparin had increased K_s_ (5.6 ± 0.9 × 10^−8^ cm^2^; *P* < .01) when thrombin was used as a trigger, correlating to larger pore size in these clots compared to control (3.5 ± 0.9 × 10^−8^ cm^2^) (Figure 4H). Permeation data and confocal images could not be obtained for NPP spiked with 0.6‐2 U/mL enoxaparin, as no clots were formed at these concentrations.

### 3.6 Fiber size following clotting initiation with TF or thrombin

**Figure 5:**
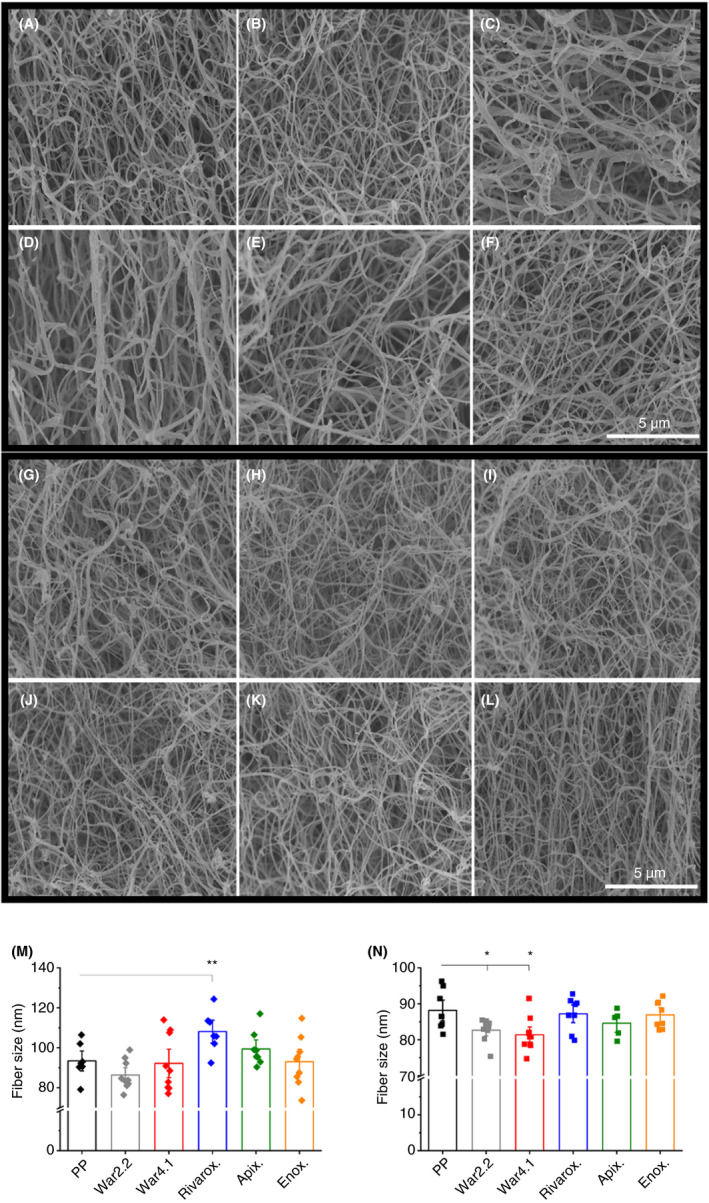
Fibrin fiber size and scanning electron microscopy (SEM) images of fully formed clots following clotting with tissue factor or thrombin. SEM images of pooled plasma (PP) (A), warfarin international normalized ratio (INR) 2.2 (B), warfarin INR 4.1 (C), PP + rivaroxaban (D), PP + apixaban (E), and PP + enoxaparin (F) clots following clotting initiation with tissue factor. SEM images of PP (G), warfarin INR 2.2 (H), warfarin INR 4.1 (I), PP + rivaroxaban (J), PP + apixaban (K), and PP + enoxaparin (L) clots following clotting initiation with thrombin. Images are a representation of one of three repeats. Scale bar represents 5 μm. Fiber size was measured from SEM images, each imaged in three different areas of the clot, at 10 000x magnification following clotting activation by tissue factor (M) and thrombin (N). Each data point corresponds to individual measurements of three replicates. Error bars correspond to ± SE. *P < .05, **P < .01

Finally, we used SEM to analyze fibrin fiber diameters. SEM images of fully formed clots, where TF was used as the clotting trigger, showed visually less dense clots formed with warfarinized plasma INR 4.1 and plasma spiked with rivaroxaban and apixaban, when compared to control (Figure 5A‐F). These observations corroborate the fiber density data obtained from laser confocal microscopy images, described above (Figure 3G). Moreover, SEM images from clots formed following clotting with thrombin also showed the same pattern determined by the average fiber counts of the corresponding samples (Figure 4G). Namely, clots from warfarinized plasma appeared denser, while clots from plasma spiked with enoxaparin were looser (Figure 5G‐L). Clots of plasma spiked with rivaroxaban, formed with TF, had increased fiber diameter size (108 ± 10 nm; *P* < .01) when compared to control (93 ± 9 nm) (Figure 5M). Increased fiber size for rivaroxaban clots correlated to their reduced density, observed by confocal microscopy (Figure 3G). Furthermore, warfarinized plasma produced clots with decreased fiber size following clotting initiation with thrombin (INR 2.2, 83 ± 3 nm; *P* < .05; and INR 4.1, 85 ± 9; *P* < .05) relative to control (88 ± 5 nm) (Figure 5N). This observation also correlated with the increased clot density for both warfarinized plasma samples, described above (Figure 4G).

## 4 DISCUSSION

Our study provides a novel systematic and comprehensive direct comparison of alterations on fibrin clot structure, caused by different classes of anticoagulants, detected with an array of different sensitive methods. In agreement with previous studies on changes in clot structure by VKAs,^29^ we find that the effects of anticoagulants on clot structure is highly dependent on the agonist used to initiate clotting. The mode of action of each anticoagulant used in this study is highlighted in a coagulation cascade schematic in the supplementary data (Figure S7). A summary of the observed changes in clot structure parameters following clotting with either TF or thrombin is shown in Table 2.

**Table 2:** Summary of changes in clot structure parameters following clotting with tissue factor or thrombin

	Tissue factor					Thrombin				
	War. 2.2	War. 4.1	Rivarox.	Apix.	Enox.	War. 2.2	War. 4.1	Rivarox.	Apix.	Enox.
MaxOD	‐	‐	‐	‐	‐	‐	‐	‐	‐	‐
Lag time	↑	↑	↑	↑	‐	‐	‐	‐	‐	↑
Av. Rate	↓	↓	‐	‐	‐	‐	↓	‐	‐	‐
Fiber density	‐	↓	↓	↓	‐	↑	↑			↓
Pore size	‐	‐	↑	↑	‐	‐	‐	‐	‐	↑
Fiber size	‐	‐	↑	‐	‐	↓	↓	‐	‐	‐

Note

‐ no significant change; ↑ significant increase, *P* ≤ .05; ↓ significant decrease, *P* ≤ .05.

Abbreviation: MaxOD, maximum absorbance.

Triggering clotting with TF or thrombin distinctly impacted on the structure of clots formed with warfarinized plasma samples and plasma containing direct FXa inhibitors or LMWH. Most striking is the fact that the most significant changes in clot structure observed with TF were seen in the rivaroxaban clots, which showed no significant changes when thrombin was used. Similarly, clotting activation with thrombin showed the most significant changes in enoxaparin clots, whereas no significant changes were observed with TF. We hypothesize that the effects observed for enoxaparin clots with thrombin (increased lag time, decreased density, and increased porosity) are directly attributable to the direct effects of this drug on thrombin, via interaction with antithrombin.^34^ Indeed, the effects of thrombin concentration on clot structure, with reduced thrombin concentrations generating less dense clots with larger pores, are well known and likely explain these findings.^25^, ^35^ TF‐bearing cells have also been shown to regulate the rate of fibrin formation from the cell surface through thrombin generation, modulating the stability and three‐dimensional structure of the clot.^26^, ^36^ In addition, elevated prothrombin concentrations have been shown to decrease fiber mass‐to‐length ratio and alter thrombin generation, when clotting initiated by TF‐bearing cells.^25^ No changes in clot structure for the FXa inhibitors were seen with thrombin, most likely due to the fact that their mode of action is upstream and largely bypassed by the exogenously added thrombin. Conversely, TF activation is upstream of factor X, allowing FXa inhibitors to demonstrate clear effects on clotting activity and clot structure. A previous study reporting less dense clots produced by endothelial cells following inhibition of TF activity further demonstrates the active role of TF on impacting clot structure.^37^ We conclude from this study that clotting initiation with TF provides more accurate depictions of the effects of DOACs and VKA on clot structure, corroborating previous reports. Effects of LMWH on clot structure, however, are sensitively detected with thrombin. Interestingly, native TEG results showed the most significant changes for both rivaroxaban and enoxaparin clots. In the absence of a trigger, and in the presence of calcium, the intrinsic pathway eventually leads to thrombin generation and clot formation. This alternative clot formation pathway to the ones targeted by the other triggers used in this study, supports the TEG findings and places further emphasis on the importance of clotting trigger selection when investigating clot structure.

Dosages of anticoagulants were chosen to reflect treatment levels, with the exception of enoxaparin for which the prophylactic dose was chosen due to the fact that therapeutic concentrations of enoxaparin (equivalent to 0.5‐1.0 anti‐FXa U/mL bidaily or 1.0‐2.0 anti‐FXa U/mL once daily)^38^ had such potent effects on clot structure as to impair analysis (Supplementary Table S1). We also observed that clot structure was influenced by different levels of VKA. This study suggests that clot structure analysis techniques, such as permeation and turbidity, have the potential to be used to assess the effects of anticoagulation drugs on patient clot phenotype.

Apparent discrepancies in data obtained by different methodologies may be attributed to the physical state of the clot, namely, hydrated fully polymerized clots (confocal and permeation), processed fully polymerized clots (SEM) and hydrated polymerizing clots (turbidity). From this, we conclude that data obtained from the different methodologies used to investigate clot structure are mostly complementary, with different structural properties such as permeability and fiber thickness being measured by each method. Our study has a number of limitations. The potential clinical application of these finding will need to be further assessed in future clinical studies using patient samples. A second limitation of this study was that confocal and SEM analyses were not performed in a blinded manner to eliminate any potential unconscious bias. A third limitation is the use of a PP spiked with anticoagulants rather than patient plasma, as patients requiring anticoagulation have a prothrombotic clot phenotype that is not reflective of the “normal” clot phenotype. A future comparison between pre‐ and post‐anticoagulation in patient plasma would provide further assessment of impact different anticoagulants have on clot parameters relevant to clinical practice, and whether anticoagulant treatment is able to revert a prothrombotic clot phenotype to a normal clot phenotype. Nevertheless, the PP used as a control in this study was obtained from a large number of individuals before they underwent a range of surgical procedures. It therefore includes individuals with and without underlying health conditions, which indicates that this PP is not entirely normal, as also indicated by a slightly above normal average fibrinogen concentration. Future studies with patient plasma would help to identify the best global clot structure method, for instance, permeation or turbidity, which do not require specialist equipment and provide quantitative data in just a few hours, with the potential to become a screening test for changes in clot structure.

Further limitations of the study include the use of a single concentration of clotting agonists and DOACs. In vivo, the concentration of free thrombin changes over the course of clot formation and thrombin concentration has been shown to directly influence clot structure, including fiber thickness and clot density.^25^, ^26^, ^35^, ^36^ Future studies correlating thrombin generation and clot structure would add value to the findings of this study. In addition, future investigations into the effect of different DOAC concentrations on alleviating prothrombotic clot phenotype could help determine a dosage to effect threshold for these drugs. Another limitation of the study was the fact that, due to warfarin’s mode of action, this could not be spiked into the same control PP as the other anticoagulants and was instead obtained from individuals treated with warfarin. We recognize that the PP from the two groups of patients on warfarin may have different properties than the PP used as a control. However, by matching the samples’ fibrinogen concentration, which is known to influence clot structure as well as feasibly possible, we ensured that the best possible comparison could be achieved.

In summary, we have shown that clot structure is significantly altered by different anticoagulants using an array of techniques and direct in vitro comparisons. In agreement with previous reports, our data show that the choice of trigger for clotting is important when investigating clot structure in samples containing different types of anticoagulants, with TF providing the most sensitive and accurate comparisons for the DOACs used in this study. Further studies are required to establish how these methods may contribute to a correlation of clot structure with clinical data either at the start of anticoagulation or over the course of treatment. Identifying changes in clot phenotype using clot structure measurements, such as permeation and turbidity, could ultimately help in the prevention of bleeding as well as thrombotic events.

## RELATIONSHIP DISCLOSURE

All authors declare no conflict of interest.

## AUTHOR CONTRIBUTIONS

JSG performed experimental work, planned and designed research, analyzed data, and wrote the article. NR conceived the study, collected blood and prepared plasma samples, performed TEG experiments, and critically reviewed the article. EMP performed experimental work and contributed to data analysis and article writing. HP advised on experimental work, and critically reviewed the article. MM conceived the study and critically reviewed the article. AG conceived the study and provided plasma samples and critically reviewed the article. RASA conceived the study, advised with experimental design and interpretation, and edited the article.

## Supporting information


**Fig S1: Additional turbidity analysis parameters of polymerising clots triggered with tissue factor**. Clotting of diluted plasma samples was triggered with tissue factor and parameters of warfarinised plasma with INR 2.22 (War2.2) and 4.11 (War4.1), and pooled plasma (PP) spiked with rivaroxaban (Rivarox.), apixaban (Apix.) or enoxaparin (Enox.) were compared against PP control. Time to 50% clotting (A) and time to maximum rate of clotting (Vmax) (B). Data points correspond to individual technical replicates. Error bars correspond to ± SE of two replicates, with three technical replicates. ****p<0.0001.

**Fig S2: Clot structure analysis of NPP spiked with higher concentrations of enoxaparin following clotting with tissue factor**. Turbidity analysis of polymerizing clots containing 0.6 U/mL, 1 U/mL or 2 U/mL enoxaparin, compared to NPP clots and buffer control (A), fibrin clot porosity (B), determined by the permeation coefficient (Ks), fibrin clot density (C), determined by confocal microscopy images. Confocal microscopy images of NPP (D) and NPP+0.6U/mL enoxaparin (E) clots; scale bar represent 50 µm. SEM images of NPP (F) and NPP+0.6U/mL enoxaparin (G) clots; scale bar represents 5 µm. Images are a representation of one of three repeats, each imaged in three different areas of the clot. Error bars correspond to ± SE of three replicates (with three technical replicates in graph A). *p<0.05, ****p<0.0001.

**Fig S3: Additional turbidity analysis parameters of polymerising clots triggered with thrombin**. Clotting of diluted plasma samples was triggered with thrombin and parameters of warfarinised plasma with INR 2.22 (War2.2) and 4.11 (War4.1), and pooled plasma (PP) spiked with rivaroxaban (Rivarox.), apixaban (Apix.) or enoxaparin (Enox.) were compared against PP control. Time to 50% clotting (A) and time to maximum rate of clotting (Vmax) (B). Each data point represents the average of three technical replicates. Error bars correspond to ± SE of four replicates, with three technical replicates. **p<0.01, ****p<0.0001.

**Fig S4: Turbidity analysis of polymerizing clots of NPP spiked with higher concentrations of enoxaparin following clotting with thrombin**. Turbidity analysis of polymerizing clots of NPP containing 0.6 U/mL, 1 U/mL or 2 U/mL enoxaparin, compared to NPP clots and buffer control. Error bars correspond to ± SE of three replicates, with three technical replicates.

**Fig S5: Tile scan images of fully formed clots following clotting with tissue factor**. 3x3 tile scan images of PP (A), warfarin INR 2.22 (B), warfarin INR 4.11 (C), PP+rivaroxaban (D), PP+apixaban (E) and PP+enoxaparin (F) obtained by confocal microscopy. Images are a representation of one of three repeats, each imaged in three different areas of the clot. Scale bar represents 100 µm.

**Fig S6: Tile scan images of fully formed clots following clotting with thrombin**. 3x3 tile scan images of PP (A), warfarin INR 2.22 (B), warfarin INR 4.11 (C), PP+rivaroxaban (D), PP+apixaban (E) and PP+enoxaparin (F) obtained by confocal microscopy. Images are a representation of one of three repeats, each imaged in three different areas of the clot. Scale bar represents 100 µm.

**Fig S7: Schematic of the coagulation cascade and how the anticoagulants used in this study inhibit fibrin clot formation**. Anticoagulants used in this study, as well as their mode of action, are shown in orange. Arrow‐heads at the end of solid or dashed lines indicate activation and solid line at the end of dashed lines indicates inhibition.

Supplementary Material
