# Supplementary figures and images for: Effect of anticoagulants on fibrin clot structure: A comparison between vitamin K antagonists and factor Xa inhibitors
Source: Res Pract Thromb Haemost. 2020 Oct 25;4(8):1269–81. doi: 10.1002/rth2.12443 (PMC7695561; doi:10.1002/rth2.12443)

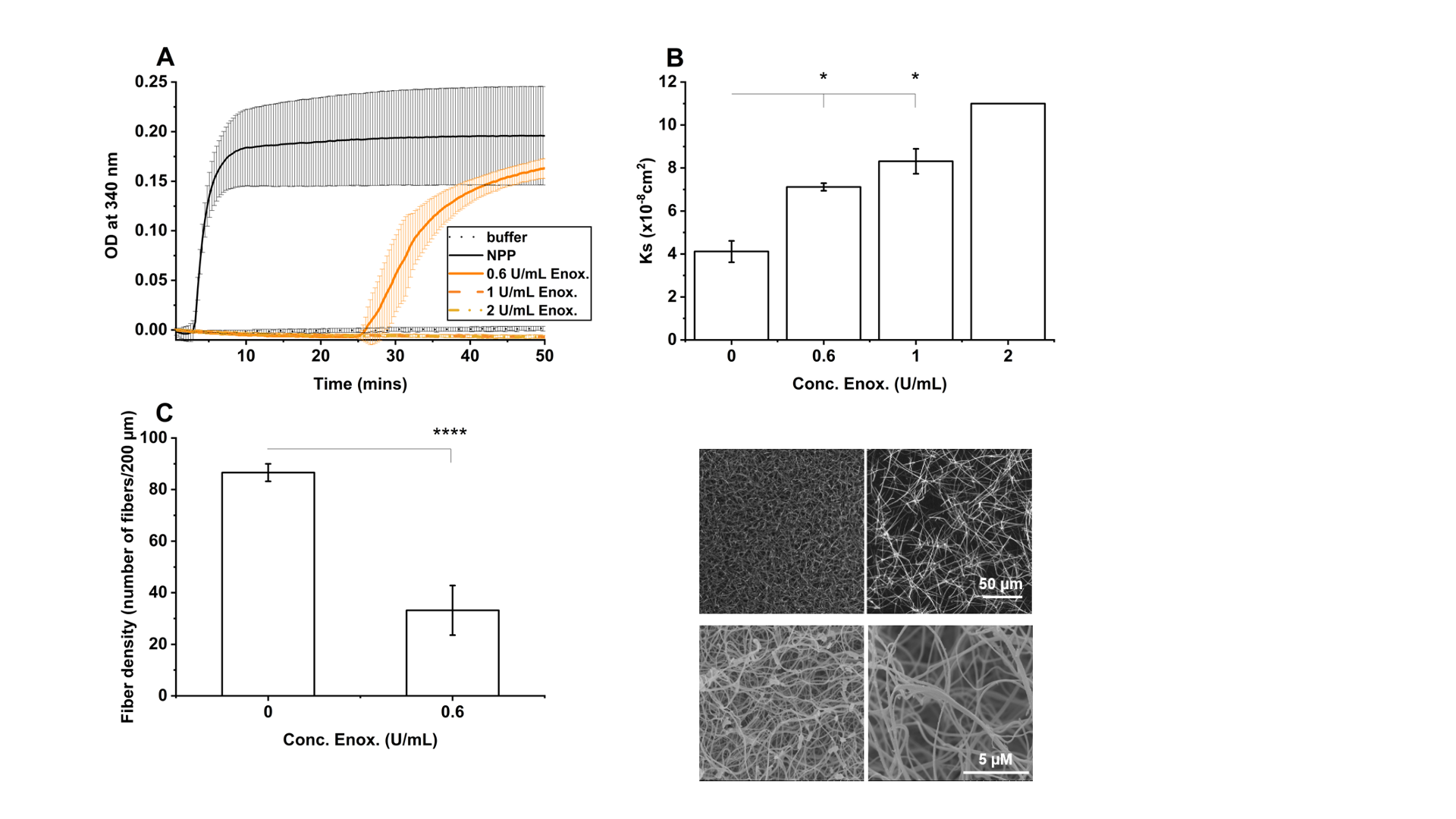

Supplement: Supplementary file 2 — Fig S2: Clot structure analysis of NPP spiked with higher concentrations of enoxaparin following clotting with tissue factor. Turbidity analysis of polymerizing clots containing 0.6 U/mL, 1 U/mL or 2 U/mL enoxaparin, compared to NPP clots and buffer control (A), fibrin clot porosity (B), determined by the permeation coefficient (Ks), fibrin clot density (C), determined by confocal microscopy images. Confocal microscopy images of NPP (D) and NPP+0.6U/mL enoxaparin (E) clots; scale bar represent 50 µm. SEM images of NPP (F) and NPP+0.6U/mL enoxaparin (G) clots; scale bar represents 5 µm. Images are a representation of one of three repeats, each imaged in three different areas of the clot. Error bars correspond to ± SE of three replicates (with three technical replicates in graph A). *p<0.05, ****p<0.0001. [file RTH2-4-1269-s002.tiff]

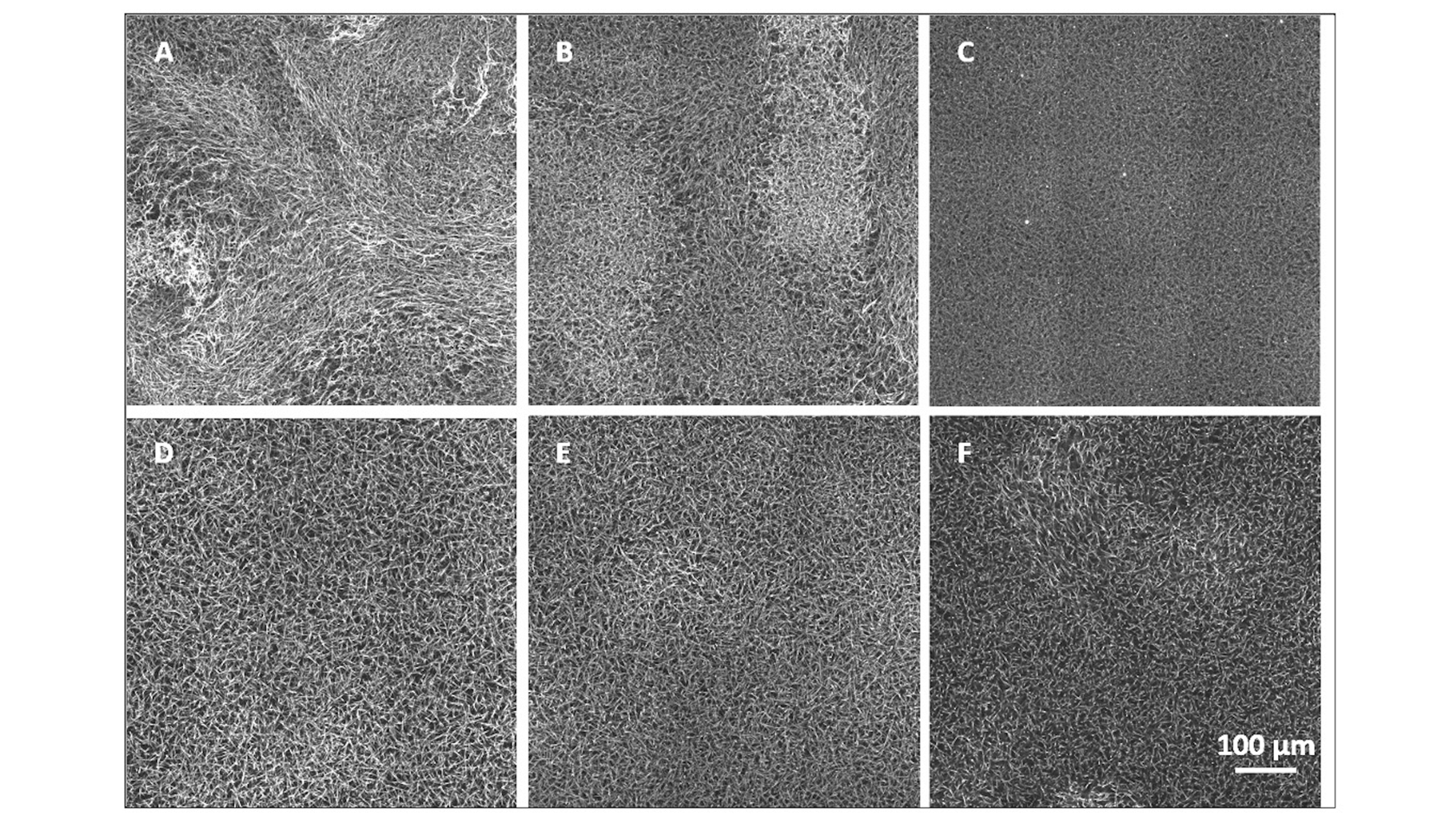

Supplement: Supplementary file 5 — Fig S5: Tile scan images of fully formed clots following clotting with tissue factor. 3x3 tile scan images of PP (A), warfarin INR 2.22 (B), warfarin INR 4.11 (C), PP+rivaroxaban (D), PP+apixaban (E) and PP+enoxaparin (F) obtained by confocal microscopy. Images are a representation of one of three repeats, each imaged in three different areas of the clot. Scale bar represents 100 µm. [file RTH2-4-1269-s005.tiff]

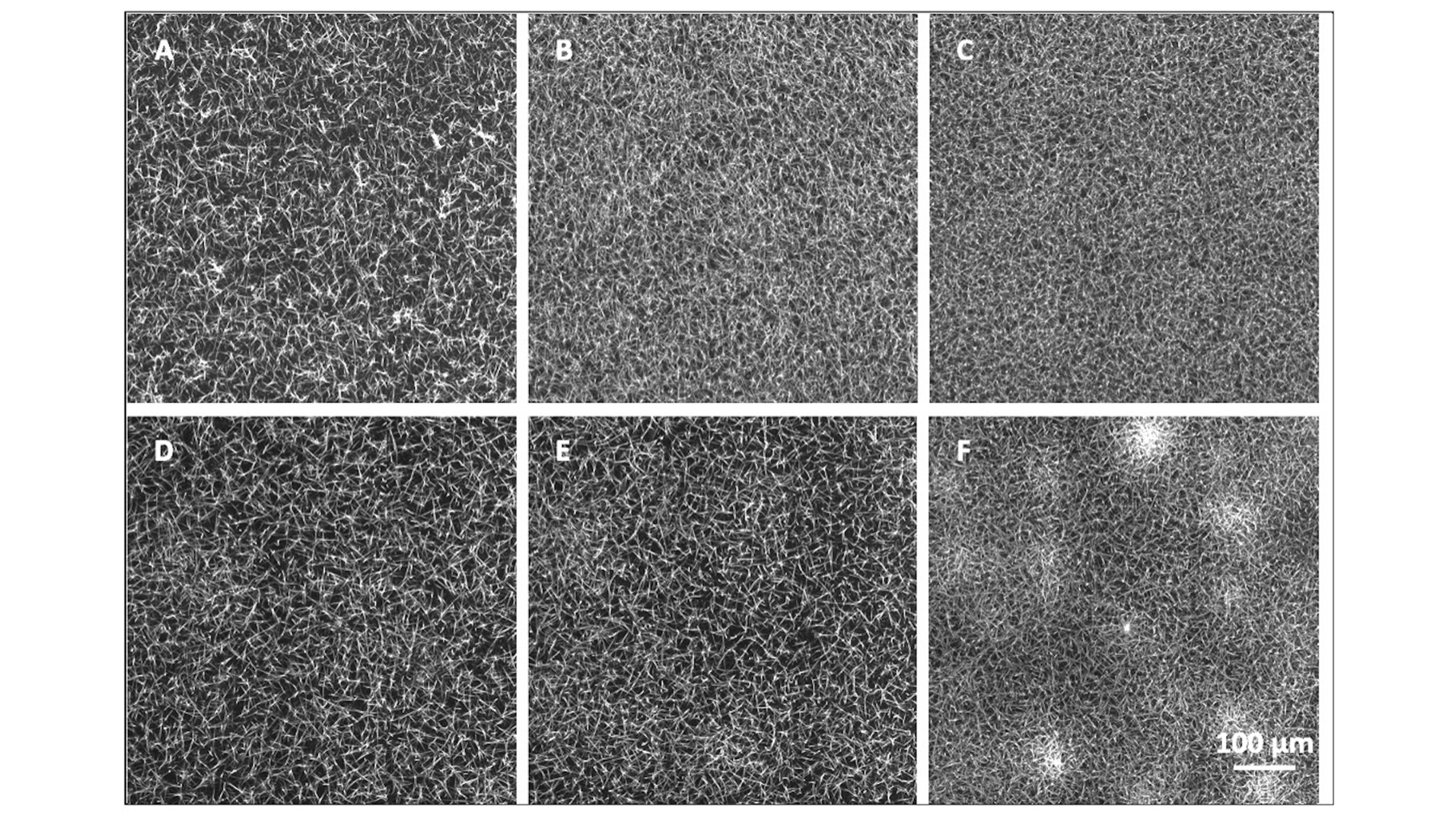

Supplement: Supplementary file 6 — Fig S6: Tile scan images of fully formed clots following clotting with thrombin. 3x3 tile scan images of PP (A), warfarin INR 2.22 (B), warfarin INR 4.11 (C), PP+rivaroxaban (D), PP+apixaban (E) and PP+enoxaparin (F) obtained by confocal microscopy. Images are a representation of one of three repeats, each imaged in three different areas of the clot. Scale bar represents 100 µm. [file RTH2-4-1269-s006.tiff]

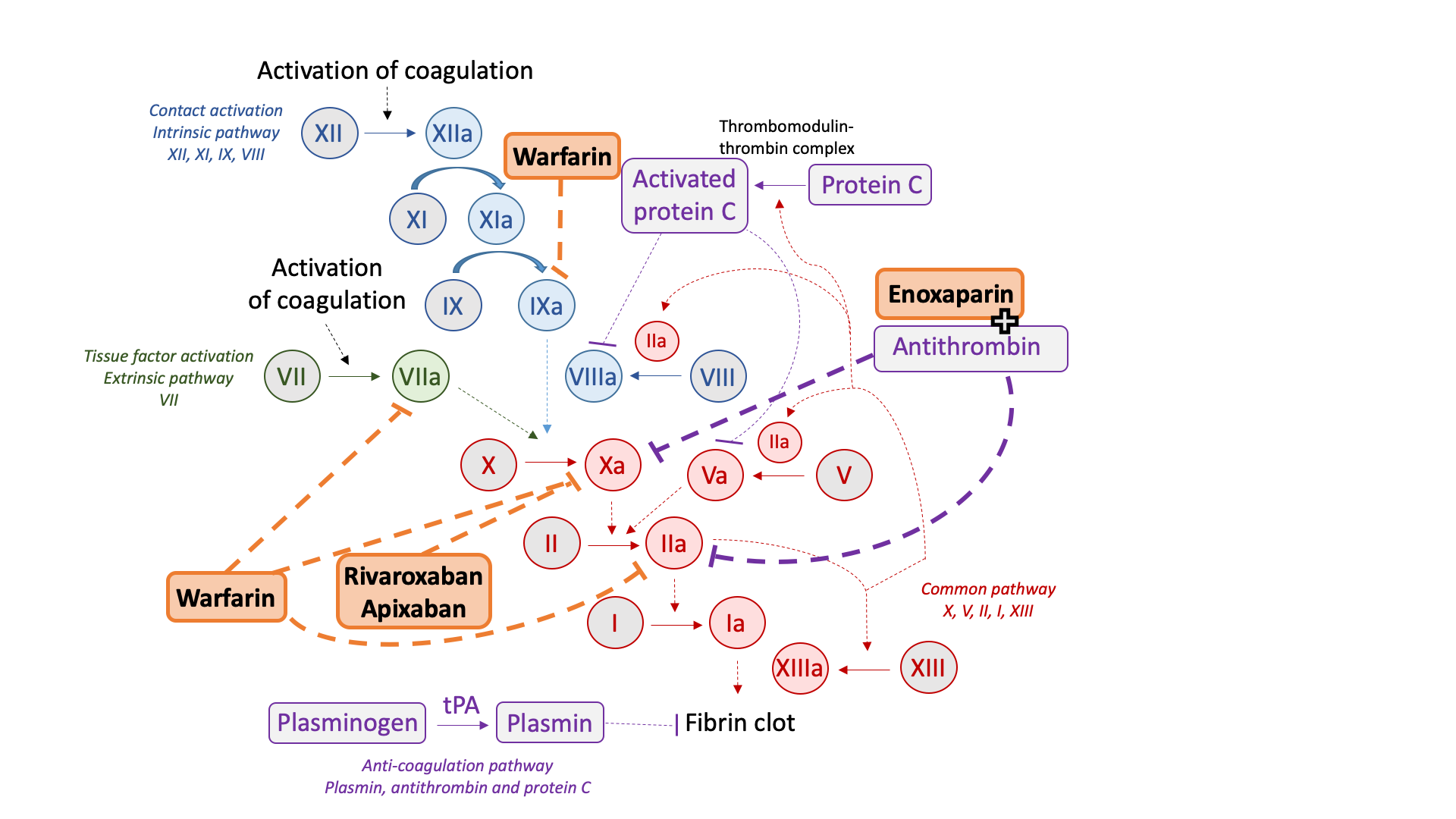

Supplement: Supplementary file 7 — Fig S7: Schematic of the coagulation cascade and how the anticoagulants used in this study inhibit fibrin clot formation. Anticoagulants used in this study, as well as their mode of action, are shown in orange. Arrow‐heads at the end of solid or dashed lines indicate activation and solid line at the end of dashed lines indicates inhibition. [file RTH2-4-1269-s007.tiff]
