# Supplementary material for: Effect of anticoagulants on fibrin clot structure: A comparison between vitamin K antagonists and factor Xa inhibitors
Source: Res Pract Thromb Haemost. 2020 Oct 25;4(8):1269–81. doi: 10.1002/rth2.12443 (PMC7695561; doi:10.1002/rth2.12443)
Supplement: Supplementary file 8 — Supplementary Material [file RTH2-4-1269-s008.docx]

Effect of anticoagulants on fibrin clot structure: a comparison between vitamin K antagonist and DOACs – supplementary table:

**Supplementary Table 1 Summary of changes in clot structure parameter in samples with higher enoxaparin concentration:**

|  | Tissue Factor | | |
| --- | --- | --- | --- |
|  | 0.6 U/mL enox. | 1 U/mL enox. | 2 U/mL enox. |
| **Lag time** | ↑ | - | - |
| **Fibre density** | ↓ | - | - |
| **Pore size** | ↑ | ↑ | ↑# |

- no clot formed
# only a single clot obtained
